# P53 Antimicrobial stewardship in Nigeria: barriers, enablers and implementation strategies in healthcare facilities

**DOI:** 10.1093/jacamr/dlaf230.060

**Published:** 2025-12-04

**Authors:** Omobolanle Margaret Abe, Rasha Abdelsalam Elshenawy

**Affiliations:** Department of Medicine, School of Health, Medicine and Life Sciences, University of Hertfordshire, Hatfield AL10 9AB, UK; Department of Medicine, School of Health, Medicine and Life Sciences, University of Hertfordshire, Hatfield AL10 9AB, UK

## Abstract

**Background:**

Antimicrobial resistance (AMR) poses a critical global health threat and disproportionately affects low and middle-income countries such as Nigeria, where inappropriate antibiotic use is widespread.^1^ Strengthening antimicrobial stewardship (AMS) is critical to tackling antimicrobial resistance (AMR).^2^ However, its implementation across Nigerian healthcare settings remains fragmented and insufficiently understood.^2^ Targeted AMS interventions are essential to address AMR, and understanding local barriers and facilitators can improve programme effectiveness, reduce AMR and enhance patient outcomes in Nigeria.^3^

**Objectives:**

To systematically review the barriers, facilitators and implementation strategies associated with AMS programmes in Nigerian healthcare facilities.

**Methods:**

A systematic search of PubMed, Scopus, Web of Science, CINAHL, Cochrane Library and Google Scholar was conducted for English-language studies published between 2015 and 2025. Inclusion criteria were studies conducted in Nigeria that reported barriers, facilitators, or strategies for AMS implementation. Study quality was assessed using the Critical Appraisal Skills Programme (CASP) checklist and the Mixed Methods Appraisal Tool (MMAT, 2018). Data extraction captured study characteristics, findings, AMS interventions and facilitators, which were synthesized through thematic and quantitative analyses.

**Results:**

A total of 844 articles were screened, of which 10 met the inclusion criteria. Barriers to AMS implementation included resistance from prescribers (10%), workload issues (20%), infrastructure gaps (40%), policy or administrative gaps (50%), resource limitations (60%) and training and education deficits, with approximately 70% of prescribers demonstrating poor AMS awareness. Facilitators included IT infrastructure (10%), institutional policies (20%), multidisciplinary teams (30%), training programmes (30%), professional expertise (70%) and leadership support (80%), emerging as central drivers of stewardship programmes in Nigerian Healthcare Facilities. Implementation strategies reported across studies involved IT and digital health integration (20%), prospective audits and feedback (30%), pre-authorization systems (30%), education and training programmes (40%), guidelines and policy development (60%), surveillance and monitoring systems (80%) and AMS teams or committee formation (80%) which provide a roadmap for improving AMS implementation in Nigerian healthcare facilities.

**Conclusions:**

Antimicrobial resistance is a challenging global health concern, particularly in Nigeria. This systematic review identified significant barriers to AMS implementation, including inadequate diagnostics, limited training and weak policies, with 70% of prescribers showing poor awareness. However, pharmacy-led initiatives and collaborative approaches show promise. Urgent investment in diagnostic infrastructure, systematic capacity building, policy reform and sustained leadership commitment are essential to improve the AMS programme effectiveness and combat antimicrobial resistance in Nigerian healthcare facilities.
